# Assessing the impact of early progressive mobilization on moderate-to-severe traumatic brain injury: a randomized controlled trial

**DOI:** 10.1186/s13054-024-04940-0

**Published:** 2024-05-22

**Authors:** Hsiao-Ching Yen, Hung-Jui Chuang, Wei-Ling Hsiao, Yun-Chen Tsai, Po-Min Hsu, Wen-Shiang Chen, Yin-Yi Han

**Affiliations:** National Taiwan University Hospital, Taipei, Taiwan

**Keywords:** Traumatic brain injury, Early mobilization, Intensive care, Intensive care units, Rehabilitation, Early ambulation

## Abstract

**Introduction:**

Traumatic brain injury (TBI) is a major cause of neurodisability worldwide, with notably high disability rates among moderately severe TBI cases. Extensive previous research emphasizes the critical need for early initiation of rehabilitation interventions for these cases. However, the optimal timing and methodology of early mobilization in TBI remain to be conclusively determined. Therefore, we explored the impact of early progressive mobilization (EPM) protocols on the functional outcomes of ICU-admitted patients with moderate to severe TBI.

**Methods:**

This randomized controlled trial was conducted at a trauma ICU of a medical center; 65 patients were randomly assigned to either the EPM group or the early progressive upright positioning (EPUP) group. The EPM group received early out-of-bed mobilization therapy within seven days after injury, while the EPUP group underwent early in-bed upright position rehabilitation. The primary outcome was the Perme ICU Mobility Score and secondary outcomes included Functional Independence Measure motor domain (FIM-motor) score, phase angle (PhA), skeletal muscle index (SMI), the length of stay in the intensive care unit (ICU), and duration of ventilation.

**Results:**

Among 65 randomized patients, 33 were assigned to EPM and 32 to EPUP group. The EPM group significantly outperformed the EPUP group in the Perme ICU Mobility and FIM-motor scores, with a notably shorter ICU stay by 5.9 days (*p* < 0.001) and ventilation duration by 6.7 days (*p* = 0.001). However, no significant differences were observed in PhAs.

**Conclusion:**

The early progressive out-of-bed mobilization protocol can enhance mobility and functional outcomes and shorten ICU stay and ventilation duration of patients with moderate-to-severe TBI. Our study’s results support further investigation of EPM through larger, randomized clinical trials.

*Clinical trial registration* ClinicalTrials.gov NCT04810273. Registered 13 March 2021.

**Supplementary Information:**

The online version contains supplementary material available at 10.1186/s13054-024-04940-0.

## Background

Traumatic brain injury (TBI) is a leading cause of neurodisability worldwide [1], with projections up to 2030 indicating its continued prominence among neurological conditions [2]. TBI is distinguished from other neurological disorders, such as stroke, by its mechanism and high velocity of injury—rapid acceleration/deceleration forces leading to axonal damage [3]—which results in both chemical and mechanical alterations within neurons. Therefore, patients with moderate to severe TBI frequently exhibit cognitive and motor impairments, which severely impact their quality of life. The prevalence of disability post-TBI is stratified by severity, with rates of 10%, 66%, and 100% for mild, moderate, and severe cases, respectively [4]. Earlier admission to post-injury rehabilitation is associated with better functional outcomes in acute care [5]. After moderate or severe TBI, adults are usually first admitted to an intensive care unit (ICU). Mobility programs in the ICU can enhance independent functional status at hospital discharge [6], highlighting the need for standardized early mobilization protocols to improve clinical outcomes and reduce healthcare costs.

Previous studies have underscored the importance of initiating rehabilitation for moderate to severe TBI as promptly as possible to enhance recovery [7–9]. “Early rehabilitation” typically refers to initiation of physical activities within two to seven days after injury or illness [10]. Moreover, integrating functional and physical activities through early mobilization has proven beneficial in ameliorating outcomes for critically ill patients [11–13]. While the significance of early mobilization after TBI is increasingly recognized, the method or precise timing for initiating such interventions specific to TBI remains indeterminate [14]. Previous studies have demonstrated the feasibility and benefits of early progressive mobilization (EPM) using ICU mobility scales, which improves the activity levels of TBI patients without adverse effects [15]. Recent research also indicates that using a modified ICU mobility scale adapted explicitly for patients with moderate to severe TBI significantly enhances their mobility by ICU discharge [16]. Moreover, post-TBI environmental enrichment has been shown to support axonal regeneration and neuroplasticity, potentially alleviating behavioral and neurological alterations [17]. Nonetheless, among other risks associated with premature upright positioning, the potential for neurological decline due to impaired cerebral autoregulation after TBI warrants prudent evaluation. This concern has catalyzed the formulation of the Early Progressive Upright Positioning (EPUP) protocol. The EPUP protocol is strategically designed to gradually transition patients to upright positions, aiming to mitigate cerebral hemodynamic fluctuations and incorporate extended initial mobilization time outside the bed [18].

The method and timing of rehabilitation training are crucial for therapeutic outcomes. However, guidelines for early mobilization of ICU-admitted TBI patients are mainly derived from the broader critical care literature [11, 13, 19–24]. Despite some evidence supporting early rehabilitation therapy for TBI recovery [16, 20], specific studies on early mobilization in moderate-to-severe TBI are scarce [16], and research on the effects of various early mobilization protocols is lacking. To address this gap, we conducted a randomized controlled study to evaluate the effectiveness of different early mobilization approaches, including their post-injury timing, for patients with moderate-to-severe TBI.

This study focused on comparing the effects of the EPM and EPUP protocols on functional outcomes of moderate-to-severe TBI patients, whose extent of brain injury precluded the near-normal neurological outcomes observed in mild TBI cases. We hypothesized that EPM (initiation of out-of-bed activities within seven days) would offer superior benefits for addressing post-TBI impairments compared to EPUP. In addition, we explored phase angles (PhAs) from bioelectric impedance analysis as potentially more precise metrics for gauging rehabilitation success, based on findings linking larger PhAs with improved functional status [25].

## Materials and methods

This study was a prospective, assessor-blinded, randomized controlled trial with two parallel groups monitored for three months. All research procedures were conducted according to the latest version of the Helsinki Declaration and The International Committee of Medical Journal Editors’ recommendations for protecting research participants. The study protocol was approved by the Institutional Review Board of the National Taiwan University Hospital (NTUH; Approval Number: 202012084RINB) and registered at ClinicalTrials.gov (Accession: NCT04810273).

### Participants

This study considered patients who presented to the trauma ICU at NTUH < 24 h after TBI. Between March 2021 and October 2023, participants meeting the study’s inclusion criteria were recruited and then randomly assigned to undergo an EPM or an EPUP protocol within three days of trauma ICU admittance. The inclusion criteria were as follows: the eligibility time window within three days of injury and an expected ICU stay of ≥ 72 h; adults with TBI aged 20–80 years [26–28]; any intracranial or extracranial bleeding in the computed tomography scan; living independently before the onset of critical illness; a GCS score of 6–13 and an injury severity score (ISS) of ≥ 16 at admission (major trauma) [29]; relatively stable respiratory status (peripheral oxygen saturation of > 92%, high mechanical ventilator setting [fraction of inspired oxygen ≤ 60%], and positive end-expiratory pressure of ≤ 10 cmH_2_O); and a stable cardiovascular system (resting heart rate of ≤ 130 bpm and not using a high-dose vasopressor > 0.2 μg kg^−1^ min^−1^) [30]. The exclusion criteria were as follows: predicted mortality within the next 24 h; palliative care; penetrating head injuries; a consistent increase in intracranial pressure (> 22 mmHg) [13]; pregnancy; uncontrolled seizure; significant bleeding suggested by systolic hypotension (< 90 mmHg) [31]; ruptured or leaking aortic aneurysm; development of acute myocardial infarction during ICU stay; rapid development of degenerative neuromuscular diseases; inability to provide informed consent; or the order that require early immobilization.

### Randomization and masking

The participants were randomized into two groups for either the EPM or the EPUP rehabilitation protocol using block randomization to balance group sizes. A 1:1 ratio and block size of four were used for allocation, with details stored in opaque, sealed envelopes managed by a research assistant. Upon patient enrollment, the envelopes were opened sequentially to assign treatments. The trial was single-blind; only the evaluating physiotherapist (PT), not involved in administering treatment, was unaware of group assignments. Due to the intervention’s nature, the blinding of patients, clinical staff, or the primary PT was not feasible. Information on adverse events was communicated by the PT to the study coordinator to maintain assessor blindness.

### Procedures

Patients in both groups received daily 30-min interventions, 5 days a week, administered by PTs alongside standard ICU care. Decisions concerning sedation (specifically, targeting a Richmond Agitation-Sedation Scale score between 0 and − 2) [32], ventilator adjustments, and thorough pre- and post-rehabilitation care were placed under the clinicians’ discretion, in adherence to the Fourth Edition of the Guidelines for the Management of Severe Traumatic Brain Injury [32, 33]. Vital signs were monitored during sessions by the primary nurse who ensured secure tube connections and performed essential nursing interventions as needed (e.g., sputum suctioning).

### Early progressive mobilization protocol (experimental group)

For the EPM group, the early progressive mobilization protocol in the trauma ICU involved initiating out-of-bed, task-specific activities as soon as possible. According to the existing literature, the initial activities undertaken out of bed may commence from 24 h to seven days after the onset [34]. In the EPM group, patients followed the early rehabilitation guidelines and the modified ICU mobility scale for progressive mobilization [16], the EPM group aimed for at least Level III mobilization (sitting on the edge of bed) within seven days after ICU admission [35]. The protocol was initiated with in-bed exercises and advanced to out-of-bed activities as soon as medically feasible, while prioritizing safety. Progression included sitting with head elevation > 60°, sitting on the bed’s edge, standing with balance exercises, and eventually walking. Each mobilization stage was contingent on the patient’s tolerance, assessed by the PT before advancing.

### Early progressive upright positioning protocol (control group)

For the EPUP group, the ICU protocol entailed progressive upright positioning, starting with 15 passive range-of-motion exercises, escalating to in-bed and active exercises, chest rehabilitation, and retraining for rolling activities (levels 0–II) [16]. Initial exercises, classified as early passive-/active-assisted, involved movements that loaded the spine and long bones. Exercise intensity was tailored based on physiological response, with adjustments made by the PT. Upright positioning began concurrently, with the patient’s upper body elevated progressively from 30° to 90° over days until ICU discharge, starting with a 15-min duration and extending up to 30 min based on tolerance. Each step in progression from level 0 to level 2 lasted a minimum of one day. Segmental trunk control training was integrated at head elevations > 60° [16]. Out-of-bed mobilization in the EPUP group commenced after seven days post-injury.

In instances where patients exhibited lower GCS scores and were thus unable to actively engage in exercises, therapists shifted their methods towards either active-assisted or passive approaches. This adjustment entailed performing repetitive activities and using sensory stimulation methods inspired by Rood’s framework. Techniques such as touch, pressure, and vibration were applied to evoke muscle responses [36]. For both groups, serious adverse events, including falls, unplanned extubation, cardiac arrest, invasive tube dislodgement, or significant arrhythmias, prompted immediate cessation of the intervention. Furthermore, the intervention was temporarily suspended due to ventilator intolerance or intervention intolerance.

### Outcome measures

The study’s primary outcome was the Perme ICU Mobility score at hospital discharge, reflecting patient mobility. Secondary outcomes included the Functional Independence Measure’s motor domain (FIM-motor) score, PhA, and skeletal muscle index (SMI) assessed with an InBody S10 system (Biospace, Seoul, Korea), alongside trauma ICU stay length and ventilation duration. These metrics were measured at baseline, ICU and hospital discharge, and three months post-injury for the FIM-motor and Perme scores, with the PhA and SMI noted at the first two instances.

The Perme ICU Mobility score, ranging from 0 to 32, evaluates mobility through 15 items for two to three points across mental status, mobility barriers, functional strength, and assistance needed and assesses bed mobility, transfer capacity, gait, and endurance, with higher scores indicating greater mobility independence. The FIM-motor score, which assesses self-care (eating, grooming, bathing, upper-body dressing, lower-body dressing, and using the toilet), sphincter control, transfers (from bed or chair to the toilet, bath, or shower), and locomotion (walking or wheelchair mobility and stair climbing), ranges from 13 to 91, with higher scores denoting greater independence.

The body composition of the participants was assessed using the InBody S10 system, which employs the bioelectric impedance analysis (BIA) method. The PhA, denoted as α, is a parameter derived from tetrapolar BIA. The PhA concept is grounded in the sinusoidal waveform of alternating current and voltage, where the voltage curve exhibits a delay compared to the current at cell membranes, resulting in a measurable phase shift, expressed as the “phase angle” in degrees. BIA calculates the geometric components of electrical impedance (Z), which encompass resistance (R), the sum of in-phase vectors, and reactance (Xc), and the sum of out-of-phase vectors. This calculation is based on the formula **Z**^**2**^** = R**^**2**^** + Xc**^**2**^. The PhA (α) is then determined using the equation PhA(°) = (Xc/R) × 180°/π, where a larger PhA indicates robust cell membranes and substantial muscle mass, signifying better cell integrity [37]. Conversely, a smaller PhA may suggest cell death, compromised cell integrity, muscle wasting, sarcopenia, or malnutrition. In our assessment of muscle mass indices, we specifically focused on the SMI, which was calculated using the InBody S10 system by dividing appendicular lean mass by the square of body height [38].

### Statistical analysis

Sample size calculation was performed using G*Power v. 3.1.9.2 (Franz Faul, Universität Kiel, Germany), based on an estimated 7-point minimal difference in Perme ICU scores between groups [39, 40]. This calculation suggested the need for 64 participants (58 plus 10% for attrition), assuming a 30% standard deviation, a medium effect size (Cohen’s d = 0.60), with an α of 0.05 and 80% power [16, 41].

Data were analyzed using the intention-to-treat approach. All statistical analyses were performed using IBM SPSS Statistics Version 22 (Armonk, NY: IBM Corp). The normality of continuous variables was tested using the Kolmogorov–Smirnov test. Results are presented as means ± standard deviations (SDs) or medians with interquartile ranges (IQRs), and categorical data as frequencies and percentages. Generalized linear mixed modeling for repeated measures, employing a linear or Gamma distribution, was used to assess changes in the FIM-motor scores, the Perme ICU Mobility scores, PhA, and SMI values. The fixed effects of interest were time, treatment group, and the time × treatment group interaction. A significant interaction indicated treatment differences in changes in outcomes over time. The random factors in each of the models were the baseline measures of the corresponding dependent variables. We used the chi-square test or Fisher’s exact test to validate categorical and ordinal variables. The independent t-test was used to assess group differences in the length of ICU stay and the duration of ventilation. Statistical significance was defined as p < 0.05 in a two-tailed test.

## Results

### Patient characteristics

**Fig. 1:**
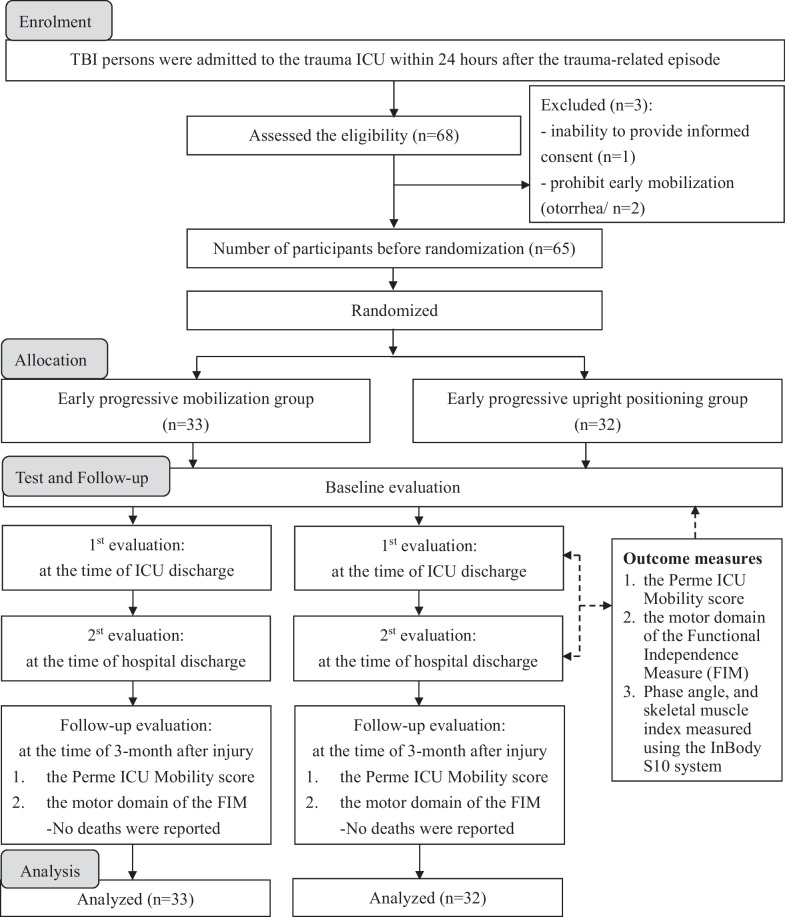
Flow diagram of patients based on CONSORT

Figure 1 presents a CONSORT diagram detailing the randomization of the 65 patients with moderate-to-severe TBI into the EPM (n = 33) and EPUP (n = 32) groups. Despite two dropouts from each group after a 3-month follow-up due to family reasons, all participants were included in the final analysis. Table 1 shows participant characteristics at enrollment, revealing no significant baseline differences between the groups except for the mean time to first mobilization post-injury (defined as the time elapsed from admission to achieving an unsupported sitting out of bed), which was significantly shorter for the EPM group (4.31 ± 1.25 days) compared to the EPUP group (12.61 ± 5.05 days; *p* < 0.001). There were no adverse events related to the early mobilization protocols during the intervention period.

**Table 1:** Patient demographic and clinical characteristics

Group	Early progressive mobilization	Early progressive upright positioning	*P* value
Number (n)	33	32	
Age, Mean ± SD	53.42 ± 20.52	50.78 ± 20.21	0.603^a^
Height, Mean ± SD	166.73 ± 8.33	163.81 ± 8.46	0.167^a^
Weight, Mean ± SD	68.43 ± 14.41	67.61 ± 10.07	0.792^a^
Male, n (%)	22 (66.7%)	21 (65%)	0.929^b^
Glasgow Coma Scale arrived in emergency room, Median (IQR)	9 (7, 13)	9 (8, 12)	0.801^c^
Charlson Comorbidity Index score, Median (IQR)	2 (0, 4)	1 (0, 3)	0.598^c^
Injury Severity Score, Median (IQR)	24 (20, 29)	29 (24, 34)	0.051^c^
Type of injury			0.181^b^
TBI, n (%)	11 (33.3%)	6 (18.8%)	
TBI with associated injury, n (%)	22 (66.7%)	26 (81.3%)	
Mechanism of injury			0.585^b^
Motor vehicle accident, n (%)	24 (72.7%)	22 (68.8%)	
Fall, n (%)	9 (27.3%)	9 (28.1%)	
Violence, n (%)	0 (0)	1 (3.1%)	
Self-harm, n (%)	0 (0)	0 (0)	
Intracranial pressure monitoring, n (%)	17 (51.5%)	14 (43.8%)	0.531^b^
External Ventricular Drainage, n (%)	1 (3%)	0 (0)	0.321^b^
Craniotomy, n (%)	10 (30.3%)	12 (37.5%)	0.540^b^
Intubation (Hypoxemia), n (%)	25 (75.8%)	29 (90.6%)	0.110^b^
Pupil dilation arrived in emergency room, n (%)	5 (15.2%)	4 (12.5%)	0.757^b^
Time to first rehabilitation intervention (days), mean ± SD	2.05 ± 1.02	2.03 ± 0.88	0.927^a^
Time to first out-of-bed mobilization (days), mean ± SD	4.31 ± 1.25	12.98 ± 6.19	< 0.001^a^*

*SD* standard deviation, *IQR* interquartile range

^a^Analyzed by Student independent t-test

^b^Analyzed by Chi-square test

^c^Analyzed by Mann–Whitney U test

^*^Significant difference between two groups (*P* < 0.05)

### Effects of early mobilization on clinical prognosis

**Fig. 2:**
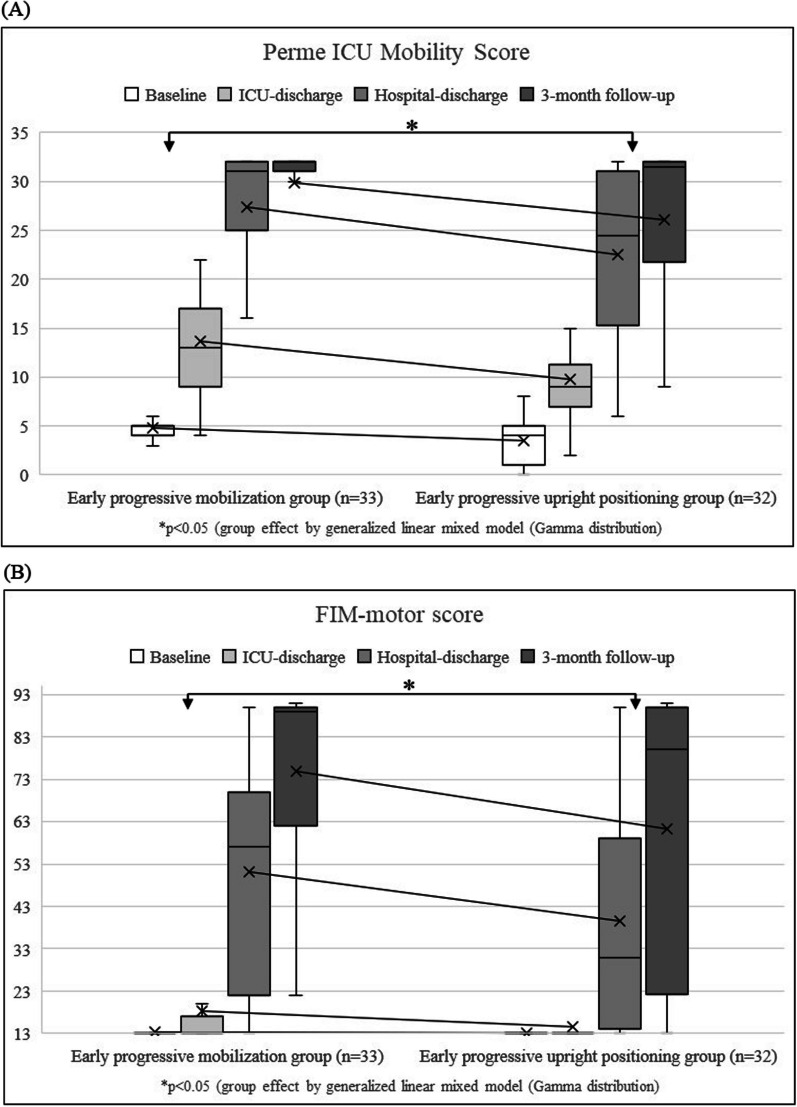
Total scores from the Perme ICU Mobility Score (**A**) and Functional Independence Measure’s motor domain (FIM-Motor) (**B**) at baseline and subsequent follow-up assessments across both groups. The graphs display medians, first and third quartiles, and range. Plus symbols (+) indicate means, connected by lines to illustrate the comparative mean values between the two groups

Figure 2 shows the total Perme ICU Mobility and FIM-motor scores at baseline, ICU discharge, hospital discharge, and three months after injury, as well as Table 2 shows SMI and PhA values at baseline, ICU, and hospital discharge for both groups. The generalized linear mixed model analysis of the Perme ICU Mobility Score, incorporating initial scores, group, time, and group × time interaction, revealed no significant difference in the interaction term (F = 2.255; *p* = 0.134). This pattern was consistent across total FIM-motor score (F = 0.233; *p* = 0.63), SMI (*p* = 0.069), and PhA (*p* = 0.813) outcomes (Fig. 2 and Table 2). However, significant group effects were observed for the total Perme ICU Mobility Score (F = 5.372; *p* = 0.021), FIM-motor score (F = 4.719; *p* = 0.031), and SMI (*p* = 0.035), but not for PhA (*p* = 0.259) (Fig. 2 and Table 2). Moreover, the EPM group experienced a significantly shorter mean ICU stay compared to the EPUP group (7.68 ± 5.64 vs. 13.6 ± 6.64 days; *p* < 0.001), as well as a shorter mean duration of ventilation (5.24 ± 7.02 vs. 11.9 ± 7.99 days; *p* = 0.001).

**Table 2:** The skeletal muscle index and phase angle measurements at baseline and each follow-up assessment in both groups

Variables	Time	Early progressive mobilization (n = 33)		Early progressive upright positioning (n = 32)		Time*Group interaction		Group effect	
		Mean	SD	Mean	SD	F value	*P* value	F value	*P* value
Skeletal muscle index	Baseline	7.28	1.3	7.47	1.26	3.345	0.069	4.499	0.035*
	ICU-discharge	7.06	1.47	6.82	1.27				
	Hospital-discharge	6.61	1.42	6.47	1.16				
Phase angle	Baseline	5.02	1.28	4.72	1.05	0.056	0.813	1.282	0.259
	ICU-discharge	4.96	1.48	5.15	1.34				
	Hospital-discharge	4.98	1.28	4.7	1.21				

FIM-motor: motor subscales of the Functional Independence Measure

*SD* standard deviation

^*^Significant difference by generalized linear mixed model (linear distribution) (*P* < 0.05)

Outcomes assessments were adjusted for baseline values

## Discussion

To the best of our knowledge, this randomized controlled trial is the first attempt to evaluate the effectiveness of both the EPM protocol and the EPUP protocol for a cohort of ICU-based patients with moderate-to-severe TBI. Crucially, the intervention commenced within seven days of the initial injury. Determination of the optimal timing for initiating out-of-bed mobilization of moderate-to-severe TBI patients is of paramount importance, as this issue continues to be a subject of debate in the medical community. Our results demonstrated that the EPM protocol, in contrast to the EPUP protocol, not only led to significantly improved total Perme ICU mobility scores but also higher FIM-motor scores and SMI values during the hospitalization period. Furthermore, the implementation of the EPM protocol was associated with reduced length of stay and duration of ventilation.

Implementation of early mobilization within seven days of ICU admission is few for patients with severe acquired brain injury [42–44]. Moreover, rare investigations have examined the effects of an early mobilization approach on physical function within the moderate-to-severe TBI population. Only a few studies have indicated that early mobilization interventions can effectively enhance severe TBI patients’ Functional Independence Measure (FIM) scores [45, 46]. Andelic et al. demonstrated that compared to a subacute and fragmented rehabilitation approach, an early and continuous rehabilitation program could lead to improved outcomes on the Glasgow Outcome Scale Extended for severe TBI patients at the 12-month post-injury stage [45]. It is important to note that the patients included in the early rehabilitation group of this study required neurointensive care for at least five days and presented with a mean ISS exceeding 35. In addition, the early rehabilitation program was founded on three distinct concepts (Affolter, Bobath, and Coombes [ABC]), which differ substantially from an early mobilization protocol employing an ICU mobility scale. Gillick et al. found that patients admitted to the trauma ICU who were involved in a structured upright sitting mobility program improved their FIM outcomes at ICU discharge compared to their time of first sitting upright [46]. In their study, patients whose monitoring equipment, such as femoral arterial line, prevented upright sitting were initially excluded, but such patients were not excluded in our study. Moreover, while the severity levels of the participants in the study were assessed based on their GCS scores within 24 h of injury, the ISS was not used as an assessment criterion. Furthermore, the patients in the retrospective group of the study were not evaluated using FIM scores, and the comparison between the two groups was primarily based on their length of stay. Yen’s research explored the potential benefits of an adapted progressive early mobilization regimen on functional mobility and out-of-bed mobility rates among patients suffering from moderate-to-severe TBI [16]. The findings indicated that employing this modified progressive early mobilization approach for patients with moderate-to-severe TBI markedly enhanced mobility at the time of ICU discharge. Nevertheless, the study utilized a retrospective and prospective pre-post intervention design without assessing follow-up outcomes post-ICU discharge. Therefore, direct comparison between our results and those of previous studies is difficult. Nevertheless, our results provide valuable insights for developing early intervention characteristics within the first seven days following injury. These characteristics can be integrated into treatment programs to enhance the effectiveness of mobilization protocols in the trauma ICU.

In the present study, the total FIM-motor and Perme ICU mobility scores of the patients in the EPM group indicated greater mobility ability and activities of daily living independence compared to the EPUP group within three months after injury. The EPUP protocol, characterized by a longer period before the first mobilization, is designed to minimize acute cerebral hemodynamic changes that may occur due to the dysfunction of cardiac baroreceptor sensitivity in individuals with moderately severe TBI [47, 48]. Its primary objective is to gradually transition bedridden patients from a lying down position to a sitting upright position, aiming to enhance breathing and circulation and reduce the risk of complications related to prolonged bed rest. The emphasis of the EPUP protocol is on the process of transitioning from lying down to sitting up, whereas early mobilization encompasses a broader spectrum of physical activities focused on functional mobility [49]. Therefore, compared to the EPUP protocol, the EPM protocol used in our study may provide a wider range of motor experiences and potentially alter the sensory integration and motor modulation and execution following a TBI. Furthermore, a previous study showed that there were no significant changes in autonomic responses, specifically heart rate variation in the frequency domain, when transitioning from a supine to a standing position in bed, during head-up tilting training in the acute phase after TBI [50]. In contrast, healthy individuals typically experience an increase in the low-frequency domain and a decrease in the high-frequency domain during such transitions, resulting in a higher low-frequency/high-frequency ratio [51]. The absence of an increase in the low-frequency domain during upright positioning in acute TBI patients might be attributed to a heightened sympathetic drive in the supine position. This elevated sympathetic activity could induce a “ceiling effect,” preventing further sympathetic activation during head-up tilting [52]. Therefore, this implies that early rehabilitation through upright positioning maybe not provide enough stimulation for acute TBI in terms of autonomic responses. Supporting these findings, pre-clinical studies have indicated that the effects of exercise after TBI can vary significantly. Motor rehabilitation strategies for TBI patients may need to be more intense and diversified to address the complex and varied outcomes associated with TBI [53].

In the present study, the SMI value of the EPM group was higher than that of the EPUP group until hospital discharge, but a similar result was not found for the PhA according to body composition analysis. Notably, both groups experienced decreases in follow-up SMI and PhA compared to their baseline values. The results for SMI suggest that early mobilization within seven days after the injury may help mitigate the loss of muscle strength. It is worth noting that previous studies indicated that resistance exercise is an effective intervention for improving outcomes of older women with low muscle mass [54, 55]. This suggests that the prevention or inhibition of sarcopenia progression through early out-of-bed mobilization may indeed be a viable approach. Our study provides evidence that early mobilization can have a positive impact on preserving skeletal lean mass. However, it is noteworthy that the PhA did not exhibit significant differences between the two groups. The PhA is often considered a marker of hydration and impaired nutritional status [56]. The limited effect of early mobilization on the PhA may suggest that it might not directly influence markers of hydration and nutritional status. However, this holistic approach could lead to a better understanding of the multifaceted impact of early mobilization on patients’ physical well-being. Future studies may need to explore patients’ nutritional intake, and other factors may provide a deeper understanding of the possible reasons for the lack of change in the PhA.

Furthermore, in our study, the duration of ventilator use and length of stay in the ICU were shorter in the EPM group than in the EPUP group. This is in line with the findings of previous meta-analyses that indicated that early mobilization is associated with several benefits related to ventilator management, including more ventilator-free days and shorter ventilator use durations [20, 44]. In addition, another previous study supported the idea that early mobilization can have a positive impact on various respiratory parameters. It showed that early mobilization improved forced vital capacity, maximum voluntary ventilation, and arterial oxygenation more effectively than breathing exercises alone [57]. However, it is important to emphasize that the effectiveness of mobilization depends on factors such as the type and timing of mobilization. Achieving an adequate mobilization dosage, considering its type and timing, is a key factor in reducing ventilator use. Our study contributes to the existing knowledge by providing further insights into this aspect. In our study, the implementation of the EPM protocol also aimed at promoting early ventilator weaning, which was associated with a reduction in the length of ICU stay. Similar results have been reported in previous studies [58–60]. Therefore, the findings of our study support the idea that early mobilization not only benefits ventilator management but also contributes to shorter ICU stays.

### Limitations

This study is constrained by several limitations. It was conducted at a single center, which affects its external validity. The modest sample size limited the statistical power to discern subtle differences between groups. Furthermore, the three-month follow-up period might have been insufficient to fully capture the long-term outcomes or any delayed effects of the interventions. Another significant limitation is the study’s exclusive focus on physical rehabilitation outcomes, which neglected the assessment of cognitive and psychological aspects, although it is recognized that evaluation of these aspects during the acute phase can be challenging. These limitations must be considered when interpreting the results of this study and in the context of future research planning.

## Conclusions

Our results advocate for the incorporation of the EPM protocol into early rehabilitation intervention for patients with moderate-to-severe TBI, demonstrating its effectiveness in improving mobility and functional independence. Furthermore, initiating early mobilization within the first week post-injury significantly decreases the length of ICU stay and the need for mechanical ventilation, emphasizing the critical role of early intervention in enhancing moderate-to-severe TBI patient recovery outcomes.

### Supplementary Information

Supplementary Material 1

Supplementary Material 2

## Data Availability

The datasets used and/or analyzed during the current study are available from the corresponding author on reasonable request.

## Abbreviations

TBI: Traumatic brain injury
GCS: Glasgow Coma Scale
ICU: Intensive Care Unit
EPM: Early progressive mobilization
EPUP: Early progressive upright positioning
PhA: Phase angle
NTUH: National Taiwan University Hospital
PT: Physiotherapist
FIM-motor: Functional independence measure’s motor domain
SMI: Skeletal Muscle Index
BIA: Bioelectric impedance analysis
SDs: Standard deviations
IQRs: Interquartile ranges
