# Supplementary material for: Assessing the impact of early progressive mobilization on moderate-to-severe traumatic brain injury: a randomized controlled trial
Source: Crit Care. 2024 May 22;28:172. doi: 10.1186/s13054-024-04940-0 (PMC11112875; doi:10.1186/s13054-024-04940-0)
Supplement: Supplementary file 1 — Supplementary Material 1 [file 13054_2024_4940_MOESM1_ESM.pdf]

FIM™ instrument

Functional Independence Measure

LEVELS		
	7 Complete Independence (Timely, Safely)<br>6 Modified Independence (Device)	**NO HELPER**
	**Modified Dependence**<br>5 Supervision (Subject = 100%+)<br>4 Minimal Assist (Subject = 75%+)<br>3 Moderate Assist (Subject = 50%+)<br><br>**Complete Dependence**<br>2 Maximal Assist (Subject =25%+)<br>1 Total Assist (Subject = less than 25%)	**HELPER**

	ADMISSION	DISCHARGE	FOLLOW-UP
**Self-Care**			
A. Eating			
B Grooming			
C. Bathing			
D. Dressing - Upper Body			
E. Dressing - Lower Body			
F. Toileting			
**Sphincter Control**			
G. Bladder Management			
H. Bowel Management			
**Transfers**			
I. Bed, Chair, Wheelchair			
J Toilet			
K. Tub, Shower			
**Locomotion**			
L. Walk/Wheelchair	W Walk<br>C Wheelchair<br>B Both	W Walk<br>C Wheelchair<br>B Both	W Walk<br>C Wheelchair<br>B Both
M. Stairs			
Motor Subtotal Score			
**Communication**			
N. Comprehension	A Auditory<br>V Visual<br>B Both	A Auditory<br>V Visual<br>B Both	A Auditory<br>V Visual<br>B Both
O. Expression	V Vocal<br>N Nonvocal<br>B Both	V Vocal<br>N Nonvocal<br>B Both	V Vocal<br>N Nonvocal<br>B Both
**Social Cognition**			
P. Social Interaction			
Q. Problem Solving			
R. Memory			
Cognitive Subtotal Score			
TOTAL FIM Score			

NOTE: Leave no blanks; enter 1 if patient not testable due to risk

Copyright © 1993 Uniform Data System for Medical Rehabilitation, a division of U B Foundation Activities, Inc

Functional Independence Measure. Copyright © 1996. Uniform Data System for Medical Rehabilitation.
All rights reserved. Reprinted with permission of UDSMR, University at Buffalo, 232 Parker Hall, 3435 Main Street, Buffalo, NY 14214.

Rehabilitation Nursing, Volume: 24, Issue: 3, Pages: 109-121
