# Supplementary figures and images for: Assessing the impact of early progressive mobilization on moderate-to-severe traumatic brain injury: a randomized controlled trial
Source: Crit Care. 2024 May 22;28:172. doi: 10.1186/s13054-024-04940-0 (PMC11112875; doi:10.1186/s13054-024-04940-0)

Perme ICU Mobility Score


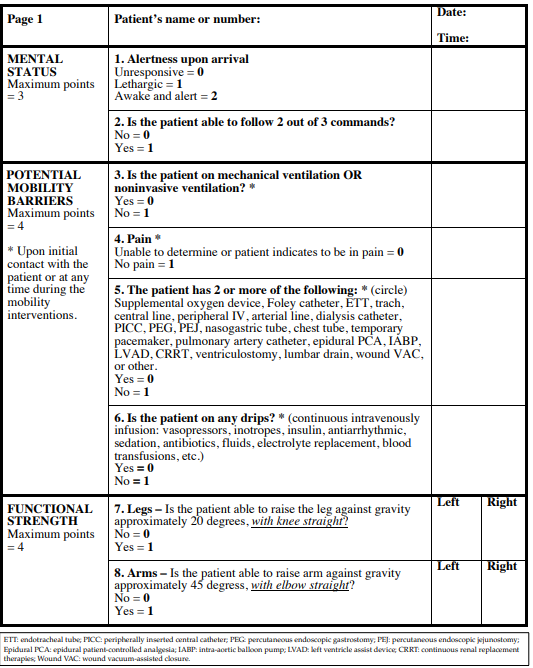


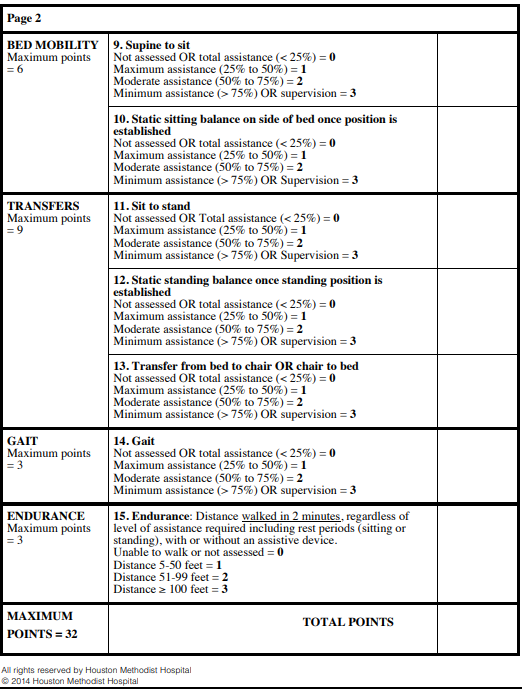


Methodist Debakey Cardiovasc J*.* 2014;10(1):41-9.

Supplement: Supplementary file 2 — Supplementary Material 2 [file 13054_2024_4940_MOESM2_ESM.doc]
